# HIV Vaccine Awareness Day: sustaining the momentum

**DOI:** 10.7448/IAS.19.1.21202

**Published:** 2016-05-18

**Authors:** Kathleen M MacQueen, Mitchell Warren

**Affiliations:** 1 Social and Behavioral Health Sciences, FHI 360, Durham, NC, USA; 2 AVAC, New York, NY, USA

What will happen to us when we get a vaccine?

The world will be a much better, safer place. To be totally honest, we *all* may be long gone, infected or not. Is that any reason to hold back?

– Bill Snow [1]

Ending the HIV epidemic requires political will, commitment to meet both scientific and implementation challenges, and overcoming human rights barriers. HIV is now a manageable chronic disease, and finding a cure is a realistic aspiration. We are also seeing an accumulation of biomedical interventions that show evidence of moderate to high efficacy for reducing the risk of HIV acquisition. The list includes antiretrovirals to prevent mother-to-child transmission as well as sexual and probably parenteral transmission, along with voluntary medical male circumcision (VMMC), oral antiretroviral prophylaxis (PrEP) and, more recently, a promising antiretroviral-containing vaginal ring to prevent HIV acquisition.

A safe, effective vaccine is not yet on this list, but nonetheless remains an essential component of any strategy to sustain long-term success against HIV. Since the virus was first identified in 1983, the search for a safe and effective vaccine has faced challenges on every front: the basic research to understand the virus and how it interacts with the human immune system, the cost of conducting clinical trials with the thousands of participants needed to statistically power a trial to evaluate efficacy, the global capacity needed to implement large trials in disadvantaged settings where HIV is most prevalent, and the ethical and human rights issues that must be addressed as a result of multiple inequities and vulnerabilities experienced by those who are recruited to participate in efficacy trials [2].

Given these challenges, some have questioned the continued allocation of resources to HIV vaccine research, arguing it is a risky investment of resources that should instead be allocated to existing HIV treatment and prevention options. This view ignores important realities. First, risky investments are the way medical science gets done. The reason we have the current array of effective treatment and prevention tools is because we invested in the research to find them, despite scientific, social and ethical scepticism. Second, we live in an evolving world, not a static one. Treatment as a path to controlling HIV will require ongoing scientific investments in a treatment pipeline to stave off viral resistance as well as significant investments in achieving the 90-90-90 treatment cascade benchmark set by UNAIDS. We need only look to the persistence of malaria and tuberculosis to gain the long view of the challenges we face with HIV. Third, biomedical interventions do not automatically translate from clinical trials to effective, scalable, sustainable programs. Experience with highly effective interventions such as VMMC and use of antiretrovirals to prevent mother-to-child transmission tells us that scaling-up PrEP and emerging approaches such as the antiretroviral-containing vaginal rings will be challenging. Last but not least, a vaccine is not redundant to other forms of prevention; it complements them. Modelling exercises suggest that even in combination, existing treatment and prevention approaches are unlikely to achieve the level of sustained impact in controlling new infection that could be achieved with the addition of a moderately effective vaccine [3]. A vaccine remains a critical tool for epidemic control.

As combination treatment and prevention programs expand, HIV vaccine trials will need to work in tandem with these proven effective methods. The more successful such programs are, the more people will need to be enrolled in vaccine trials in order to evaluate efficacy. This is the kind of challenge we long for, as it signifies reduced incidence at the community level. It is going to be complicated but not impossible. The first and only HIV vaccine trial to demonstrate proof of concept was the RV144 trial, with vaccine efficacy estimated at 31.2% (95% CI, 1.1 to 52.1; *p=*0.04) [4]. The trial provided valuable insights into how a vaccine may work to prevent HIV infection [5]. The trial enrolled 16,402 adults in two provinces in Thailand and had an observed incidence rate of 0.28 per person year of observation in the placebo arm. The RV144 trial thus also demonstrated proof of concept that large community-based HIV vaccine trials can be successfully implemented.

Now, the HIV vaccine field is at one of its most exciting points. The HIV vaccine pipeline is increasingly diverse, with more than 30 vaccine clinical trials underway, testing a variety of candidates and vaccine concepts. Importantly, four major large-scale trials are either now in the field or will be launched in the coming year, making this the most dynamic time in HIV vaccine history.

HIV Vaccine Awareness Day grew out of the recognition that prevention, like treatment, requires advocacy. The first HIV Vaccine Awareness Day was held on 18 May 1998 to recognize and thank the thousands of volunteers, community members, health professionals and scientists who are working together to find a safe and effective HIV vaccine. As the science has grown more complex over the past 18 years, sustaining momentum in the search for an HIV vaccine requires transparent and ongoing dialogue with the diverse range of global HIV prevention and treatment stakeholders. It is not just the virus we are fighting. The HIV epidemic is embedded within challenges at all levels, including gender dynamics at the interpersonal; stigma, marginalization and human rights abuses at various levels; and economic disparities from the local to the global. Community engagement and participation in decision-making about HIV vaccine trials remains even more critical. Active participation of those at risk for HIV, who may be highly stigmatized or criminalized in their home communities, is needed, and their important contributions as volunteers must be recognized and their wellbeing protected. This is no different than the requirements for advancing research on a cure for HIV [6], on new methods of treatment and prevention such as injectable antiretrovirals; or on implementation research on treatment, PrEP and VMMC. As with the science of developing an HIV vaccine, these are challenges to be confronted and overcome, and not reasons to give up.

As with every other advance made in the fight against HIV, finding a safe and effective vaccine requires solid science, persistent advocacy and sustained engagement of multiple stakeholders. Yes, finding a safe and effective vaccine will continue to be hard and expensive and take a long time. In the history of HIV, that has never been a reason to hold back.
