# Erratum

**DOI:** 10.7448/IAS.19.1.21252

**Published:** 2016-06-09

Regarding the paper ‘HIV Vaccine Awareness Day: sustaining the momentum’ by Kathleen M MacQueen, Mitchell Warren.

Published in *Journal of the International AIDS Society*, Citation: MacQueen KM and Warren M. *Journal of the International AIDS Society* 2016, **19**:21202. http://www.jiasociety.org/index.php/jias/article/view/21202 ∣ http://dx.doi.org/10.7448/IAS.19.1.21202

Reference 6 is missing in the reference list, and should be cited in the text according to the following: “This is no different than the requirements for advancing research on a cure for HIV [6], on new methods …”.

The correct reference list is displayed below.
